# On-line Coupling of Aptamer Affinity Solid-Phase Extraction and Immobilized Enzyme Microreactor Capillary Electrophoresis-Mass Spectrometry for the Sensitive Targeted Bottom-Up Analysis of Protein Biomarkers

**DOI:** 10.1021/acs.analchem.1c03800

**Published:** 2022-05-02

**Authors:** Hiba Salim, Roger Pero-Gascon, Estela Giménez, Fernando Benavente

**Affiliations:** Department of Chemical Engineering and Analytical Chemistry, Institute for Research on Nutrition and Food Safety (INSA·UB), University of Barcelona, 08028 Barcelona, Spain

## Abstract

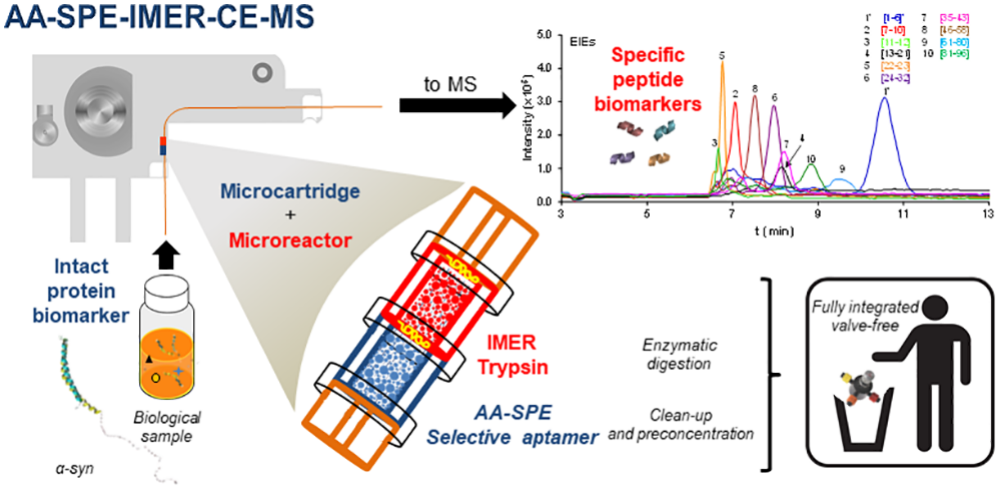

In this paper, we present a fully integrated valve-free method for the sensitive targeted bottom-up analysis of proteins through on-line aptamer affinity solid-phase extraction and immobilized enzyme microreactor capillary electrophoresis-mass spectrometry (AA-SPE-IMER-CE-MS). The method was developed analyzing α-synuclein (α-syn), which is a protein biomarker related to different neurodegenerative disorders, including Parkinson’s disease. Under optimized conditions, on-line purification and preconcentration of α-syn, enzymatic digestion, electrophoretic separation, and identification of the tryptic peptides by mass spectrometry was achieved in less than 35 min. The limit of detection was 0.02 μg mL^–1^ of digested protein (66.7% of coverage, i.e., 8 out of 12 expected tryptic peptides were detected). This value was 125 and 10 times lower than for independent on-line digestion by IMER-CE-MS (2.5 μg mL^–1^) and on-line preconcentration by AA-SPE-CE-MS (0.2 μg mL^–1^). The repeatability of AA-SPE-IMER-CE-MS was adequate (at 0.5 μg mL^–1^,% RSD ranged from 3.7 to 16.9% for peak areas and 3.5 to 7.7% for migration times of the tryptic peptides), and the modified capillary could be reused up to 10 analyses with optimum performance, similarly to IMER-CE-MS. The method was subsequently applied to the analysis of endogenous α-syn from red blood cell lysates. Ten α-syn tryptic peptides were detected (83.3% of coverage), enabling the characterization and localization of post-translational modifications of blood α-syn (i.e., *N*-terminal acetylation).

Research of protein biomarkers of physiological and pathological processes drives a great part of human proteomics studies in biological samples, as a potential tool to understand biological mechanisms and improve prevention, diagnosis, prognosis or therapeutic treatments of diseases.^1−3^ However, proteome diversity and sample matrix complexity pose a great analytical challenge, especially when dealing with proteins presenting many proteoforms,^4,5^ low abundance proteins, and limited volumes of sample.^6,7^ Nowadays, mass spectrometry (MS)-based proteomics is the gold standard in the field.^1−3^ In most cases, the bottom-up analysis of proteins, which requires digestion into peptides, is preferred over intact protein analysis.^1^ The bottom-up approach offers multiple advantages, including increased chromatographic/electrophoretic separation and MS ionization efficiencies for complex peptide mixtures, and has prompted the development of a wide variety of bioinformatic resources to ease data interpretation in shotgun untargeted bottom-up analysis for global proteome profiling. As an alternative to shotgun proteomics, in the last years, targeted proteomics is generating a great interest for the straightforward, accurate, and sensitive measurement of specific protein biomarkers from characteristic signatures of surrogate peptide fragments.^2,8−10^ In targeted proteomics, selectivity is mostly entrusted to the accuracy and resolution of the mass spectrometer, which measures a target list of peptide ions in a sensitive MS or tandem MS (MS-MS) mode. Anyway, when dealing with complex biological samples, an appropriate sample purification or fractionation before protein digestion ensures the best method performance because it minimizes the chance of undesirable sample matrix effects (e.g., ion suppression or poor peak shape).^6,7^

Capillary electrophoresis-mass spectrometry (CE-MS) is a very suitable technique for the highly efficient electroseparation and identification of charged biomolecules, including peptides and proteins.^11−13^ Over the years, different strategies have been described to decrease the limits of detection (LODs) in CE-MS,^14−17^ which are compromised due to the reduced sample volume injected to obtain optimum separations, as in other microscale chromatographic techniques applied in MS-based proteomics.^18,19^ An extremely versatile and efficient alternative is on-line solid-phase extraction capillary electrophoresis-mass spectrometry (SPE-CE-MS).^16,17^ In a recent study,^20^ we demonstrated that on-line aptamer affinity SPE-CE-MS (AA-SPE-CE-MS) can be an excellent alternative to improve detection sensitivity of CE-MS for intact protein biomarkers, while minimizing sample handling and increasing analytical throughput. A microcartridge containing a sorbent with an aptamer against α-synuclein (α-syn), which is a biomarker of Parkinson’s disease,^21−23^ was integrated near the inlet of the separation capillary. The system was operated without valves and allowed cleaning-up and preconcentrating up to 100-fold the target protein from a large volume of sample (<100 μL). Compared to the typical aptamer- or antibody-based biosensors or bioassays, a great advantage of AA-SPE-CE-MS is that the electrophoretic separation and the selectivity of the MS detection prevented the possibility of a false positive or an erroneous quantification of the target protein.^24,25^ In addition, AA sorbents have advantages over immunoaffinity sorbents,^26^ including compatibility with acidic background electrolyte (BGE) in SPE-CE-MS for optimum detection sensitivity in positive electrospray ionization (ESI+) mode, in contrast to the neutral BGEs required to avoid antibody denaturation.^27,28^ In a different study,^29^ we demonstrated that similar valve-free systems can be set up for the bottom-up analysis of proteins by immobilized enzyme microreactor capillary electrophoresis-mass spectrometry (IMER-CE-MS). In this case, a microreactor packed with immobilized trypsin particles was used for the on-line enzymatic digestion of β-lactoglobulin, α-casein, β-casein, κ-casein, and *Escherichia coli* whole cell lysates followed by separation and characterization of the tryptic peptides. Results were comparable to the off-line digestion with trypsin in solution or immobilized trypsin, while IMER-CE-MS enabled reducing protein sample volume, shortening digestion times, minimizing sample handling, and reusing microreactors containing very limited amounts of trypsin particles.

This study describes for the first time a fully integrated valve-free on-line aptamer affinity solid-phase extraction and immobilized enzyme microreactor capillary electrophoresis-mass spectrometry (AA-SPE-IMER-CE-MS) method for targeted proteomics. The method was developed and validated for the analysis of blood α-syn, as a proof-of-concept of its great potential for the sensitive, reliable, and high-throughput targeted analysis of protein biomarkers from specific surrogate peptides. The fully integrated method allowed better LODs compared to the analysis of intact α-syn by AA-SPE-CE-MS as well as a more detailed characterization, including localization of characteristic post-translational modifications (PTMs), by incorporating the benefits of enzymatic digestion.

## Experimental Section

### Materials and Reagents

All chemicals used in the preparation of the BGE and the rest of solutions were of analytical reagent grade or better. Acetic acid (HAc, glacial), ammonium hydroxide (NH_4_OH) (25%), formic acid (HFor, 99.0%), and sodium hydroxide (≥99.0%) were supplied by Merck (Darmstadt, Germany). Ammonium bicarbonate (LC-MS grade) was purchased from Sigma-Aldrich (St. Louis, MO). Propan-2-ol (LC-MS) was purchased from Scharlau (Barcelona, Spain). Water (LC-MS grade) was supplied by Fisher Scientific (Loughborough, U.K.). Particles with immobilized trypsin were provided by Promega (Madison, WI).

The DNA aptamer M5-15^30^ modified with a C6 spacer arm terminated by 5′amino (M5-15-5′, 66-mer, *M*_r_ = 20 690) and purified by HPLC, was purchased from Integrated DNA Technologies (Coralville, IA). LOABeads AffiAmino magnetic beads (MBs) of 45–165 μm diameter were purchased from Lab on a Bead (Uppsala, Sweden).

### Electrolyte Solutions, Sheath Liquid, Protein Standards, and Blood Samples

The BGE containing 50 mM HAc and 50 mM HFor (pH 2.3) was filtered through a 0.20 μm nylon filter (Macherey-Nagel, Düren, Germany). The sheath liquid solution consisted of a mixture of 60:40 (v/v) propan-2-ol:water with 0.05% (v/v) of HFor and was delivered at a flow rate of 3.3 μL min^–1^ by a KD Scientific 100 series infusion pump (Holliston, MA). The sheath liquid and the BGE were degassed for 10 min by sonication before use.

Recombinant human α-syn expressed in *Escherichia coli* was purchased from Analytik Jena (Jena, Germany). The solution provided by the manufacturer (5000 μg mL^–1^ in phosphate buffered saline (PBS)) was aliquoted and stored in a freezer at −20 °C. Aliquots were thawed before use and working standard solutions were prepared by diluting in water. These solutions were stored in the fridge at 4 °C when not in use.

Human blood from a healthy volunteer was processed, and research was conducted following standard operation procedures with appropriate approval of the Ethical and Scientific Committees of the University of Barcelona.

### Preparation of Thermo-Enriched Red Blood Cell Lysates

Thermo-enriched red blood cell (TE RBC) lysates were prepared from fresh blood as described in our previous study.^20^

Under optimized conditions, compounds of low *M*_r_ were removed from the TE RBC by passage through 10 000 *M*_r_ cut-off (MWCO) cellulose acetate filters (Amicon Ultra-0.5, Millipore). A total of 250 μL of sample was centrifuged at 12 000*g* for 10 min, and the residue was washed three times with 150 μL of water for 10 min in the same way. The final residue was recovered by inverting the upper reservoir in a vial and spinning once more at a reduced centrifugal force (3 min at 1000*g*). Sufficient water was added to adjust the final volume to 250 μL.

### Apparatus

pH measurements were made with a Sension+ PH3 potentiometer and an electrode 50 14 T (Hach Lange Spain S.L.U., Barcelona, Spain). Agitation was performed with a Vortex Genius 3 (Ika, Staufen, Germany). Centrifugal filtration was carried out in a Mikro 220 centrifuge (Hettich Zentrifugen, Tuttlingen, Germany). Incubations were carried out in a TS-100 thermoshaker (Biosan, Riga, Latvian Republic). A neodymium cube magnet (12 mm, N48) was supplied by Lab on a Bead.

All analyses were performed in a 7100 CE system coupled with an orthogonal G1603 sheath-flow interface to a 6220 oa-TOF LC/MS spectrometer equipped with ChemStation and MassHunter softwares (Agilent Technologies, Waldbronn, Germany). The TOF mass spectrometer was operated in ESI+ mode, and the optimized parameters are presented in the Supporting Information.

Fused silica capillaries were supplied by Polymicro Technologies (Phoenix, AZ). All capillary rinses were performed flushing at 930 mbar. New capillaries were activated flushing off-line with water (5 min), 1 M NaOH (15 min), water (15 min), and BGE (10 min) to avoid the unnecessary contamination of the MS system.

### IMER-CE-MS

Construction of the double frit particle-packed microreactor and the optimized method for IMER-CE-MS were based on a previous study.^29^ First, a polymeric frit was placed at one of the ends of the microreactor body (0.7 cm total length (*L*_T_) × 250 μm internal diameter (i.d.) × 365 μm outer diameter (o.d.) fused silica capillary) and this side was connected to the inlet of the separation capillary (7.5 cm *L*_T_ × 75 μm i.d. × 365 μm o.d. fused silica capillary) using a plastic sleeve. Second, the microreactor was filled with the immobilized enzyme particles applying vacuum during 10 s. The packing was checked under an optical microscope (100×), and the procedure was repeated until the microreactor was completely packed. Then, another polymeric frit was introduced at the free end of the microreactor, which was finally connected to the outlet of the separation capillary (64.5 cm *L*_T_ × 75 μm i.d. × 365 μm o.d. fused silica capillary) using another plastic sleeve. Before the analyses, the IMER-CE capillary was checked for abnormal flow restriction, flushing with water and BGE with a syringe, and applying a separation voltage of +25 kV for 15 min. All analyses were performed at 37 °C.

The IMER-CE capillary was conditioned flushing with BGE for 2 min. Two plugs of digestion buffer (10 mM NH_4_HCO_3_, pH 7.9) were injected at 50 mbar for 8 s (∼40 nL,^31^ i.e., ∼1 cm) before and after the protein sample, which was injected in digestion buffer at 50 mbar for 15 s (∼80 nL,^31^ i.e., ∼2 cm). Then, protein sample was slowly pushed introducing BGE at 5 mbar for 600 s (∼325 nL,^31^ i.e., ∼7 cm) to ensure enough time for the protein digestion through the microreactor, before applying the separation voltage (+25 kV). Between consecutive analyses, the capillary was flushed with BGE (5 min) and water (5 min) to avoid carry-over.

### AA-SPE-IMER-CE-MS

Aptamer affinity-magnetic beads (AA-MBs) were prepared as described in a previous study with minor modifications,^20^ specifically bovine serum albumin (BSA) was used as blocking agent to reduce nonspecific interactions instead of ethanolamine. A 200 μL aliquot of MBs solution was vortexed and the supernatant was removed after magnetic separation, using a cube magnet to sediment the particles (20 μL of sedimented MBs). The MBs were washed using 200 μL of PBS with 0.1% Tween 20 (PBS-T), the supernatant was removed by magnetic separation, and the MBs resuspended with the same volume of PBS-T. A volume of 10 μL of activation buffer was added, and the MBs were moderately shaken for 15 min at room temperature. The supernatant was removed by magnetic separation, and the MBs were washed with 200 μL of PBS-T and resuspended with 150 μL of PBS-T. A volume of 50 μL of the DNA aptamer M5-15-5′amino dissolved in PBS (100 μmol L^–1^) was then added to the MBs suspension. The mixture was moderately shaken for 40 min at room temperature. The supernatant was removed, and the AA-MBs were subsequently washed three times with 200 μL of PBS and resuspended with the same volume of PBS. The remaining reactive groups on AA-MBs were blocked adding 5% BSA in PBS-T, and the mixture was moderately shaken for 2 h at 37 °C. Finally, the supernatant was removed, and the AA-MBs were subsequently washed three times with 200 μL of PBS. The AA-MBs were stored in PBS with 20% (v/v) ethanol at 4 °C when not in use.

**Figure 1:**
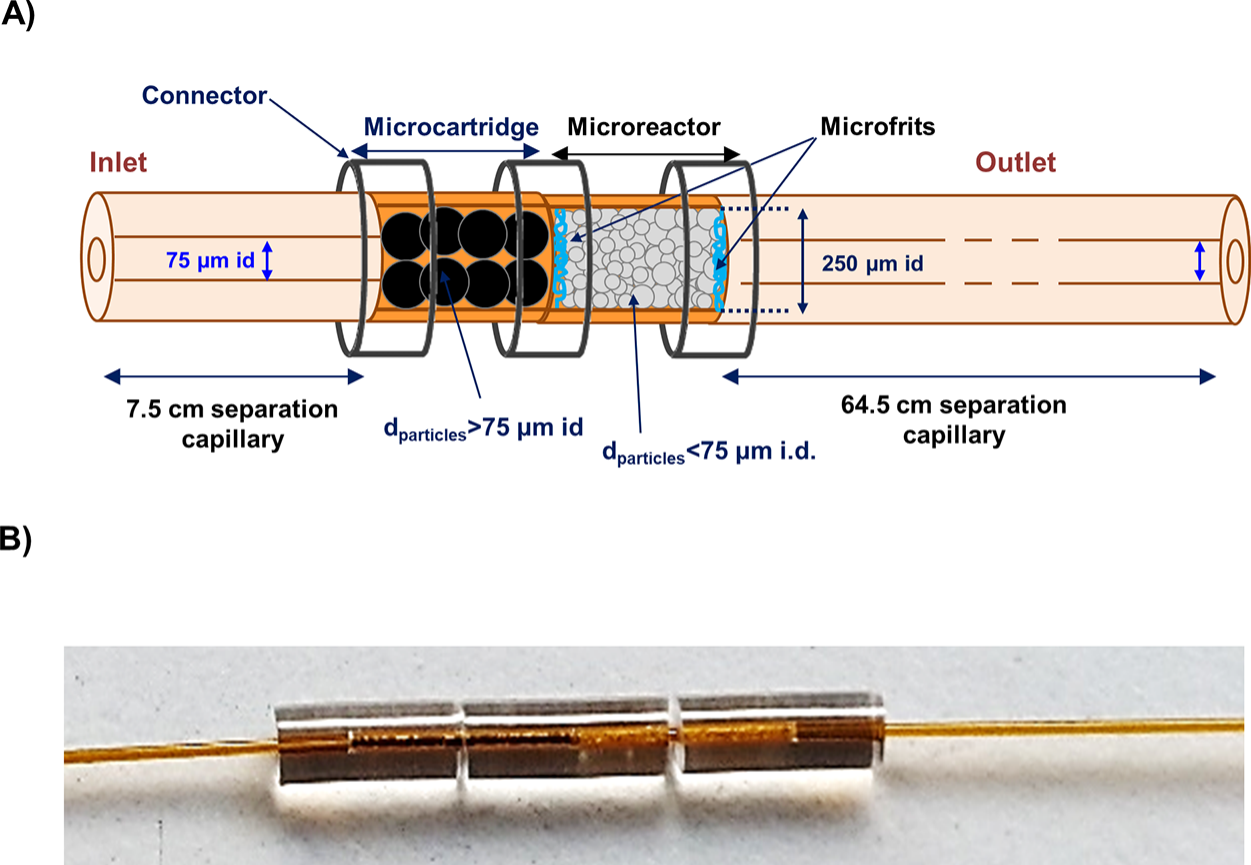
(A) Schematic representation and (B) picture under the optical microscope (100×) of a modified capillary with a microcartridge and a microreactor for AA-SPE-IMER-CE-MS.

For construction of the AA-SPE-IMER-CE capillary, the separation capillary (72 cm total length (*L*_T_) × 75 μm internal diameter (i.d.) × 365 μm outer diameter (o.d.) fused silica capillary) was activated and cut into two pieces of 7.5 cm (inlet) and 64.5 cm (outlet). First, an IMER microreactor (0.5 cm *L*_T_ × 250 μm i.d. × 365 μm o.d. fused silica capillary) was prepared as in IMER-CE-MS in one of the ends of the outlet of the separation capillary. Separately, the AA-SPE microcartridge body (0.5 cm L_T_ × 250 μm i.d. × 365 μm o.d. fused silica capillary) was connected with a plastic sleeve to a disposable capillary (5 cm *L*_T_ × 75 μm i.d. × 365 μm o.d. fused silica capillary) to be completely filled by vacuum with AA-MBs. Then, after removing the disposable capillary, the AA-SPE microcartridge was connected with plastic sleeves to the inlet of the separation capillary and the IMER microreactor (Figure 1). Before the analyses, the AA-SPE-IMER-CE capillary was checked for abnormal flow restriction, flushing with water and BGE with a syringe, and applying a separation voltage of +25 kV for 15 min. All analyses were performed at 37 °C.

Under optimized conditions, AA-SPE-IMER-CE-MS capillaries were conditioned flushing with BGE for 2 min followed by sample introduction at 930 mbar for 5 min (∼30 μL^31^). A final flush with BGE for 2 min removed nonretained molecules from the AA sorbent and filled the capillary before the elution, digestion, and separation. All these initial steps were performed with the nebulizer gas and the ESI capillary voltage switched off to prevent the entrance of contaminants into the MS. Then, both were switched on and a small volume of eluent with 100 mM NH_4_OH (pH 11.2) was injected at 50 mbar for 40 s (∼220 nL,^31^ i.e., ∼5 cm). The eluent was slowly pushed introducing BGE at 5 mbar for 600 s (∼325 nL,^31^ i.e., ∼7 cm) to ensure enough time for the protein digestion through the microreactor, before applying the separation voltage (+25 kV). Between consecutive analyses, the capillary was flushed with water for 1 min, eluent was injected at 50 mbar for 40 s, and the capillary was flushed again with water for 1 min. No carry-over was observed between consecutive analyses when this washing sequence was applied.

### Quality Parameters

The details regarding LOD, repeatability of peak area and migration time, linearity, and modified capillary lifetime in IMER-CE-MS and AA-SPE-IMER-CE-MS are given in the Supporting Information.

## Results and Discussion

### Bottom-up Analysis of Recombinant α-syn by IMER-CE-MS

**Figure 2:**
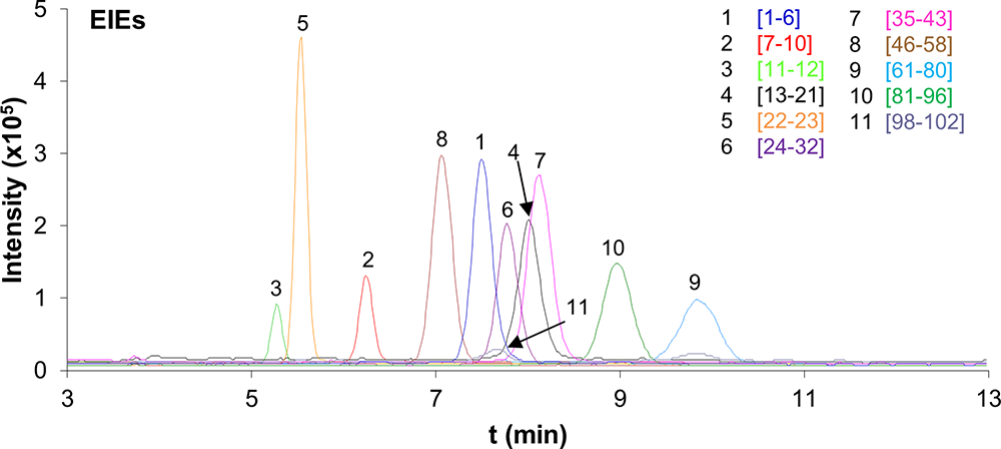
Extracted ion electropherograms (EIEs) of the tryptic peptides for the analysis by IMER-CE-MS of a 10 μg mL^–1^ recombinant α-syn standard. Conditions: microreactor (0.7 cm *L*_T_ × 250 μm i.d.), separation capillary (72 cm *L*_T_ × 75 μm i.d.) and BGE (50 mM HAc and 50 mM HFor, pH = 2.3). Sample dissolved in digestion buffer (10 mM NH_4_HCO_3_, pH 7.9), injected at 50 mbar for 15 s between two plugs of digestion buffer (50 mbar, 8 s) and pushed with BGE at 5 mbar for 600 s. Digestion-separation temperature 37 °C and separation voltage +25 kV. Data extraction considering the *m*/*z* of the most abundant molecular ions (Table 1) and a window of ±20 ppm.

In a recent study,^29^ we demonstrated the good performance of IMER-CE-MS using immobilized trypsin particles for the bottom-up analysis of bovine milk proteins and complex protein mixtures of *Escherichia coli* whole cell lysates. This method was investigated here for the analysis of recombinant α-syn. Table 1 shows the peptide sequence and the theoretical mass-to-charge (*m*/*z*) ratio of the molecular ions of free α-syn tryptic peptides detected using a BGE of 50 mM HAc and 50 mM HFor (pH 2.3). Figure 2 shows the extracted ion electropherograms (EIEs) and Table 1A the peak area and the migration time values as well as the percentages of relative standard deviation (% RSD, *n* = 3), of the tryptic peptides for a 10 μg mL^–1^ recombinant α-syn standard. At this protein concentration, 11 out of 12 expected peptides were detected (91.7% of coverage). Only the largest peptide [103-140] (Table 1) was not detected probably because of its worse ionization efficiency. Repeatability was adequate with a % RSD ranging from 0.2 to 18.2% for peak areas and 1.2 to 2.1% for migration times, the microreactor could be reused up to 10 analyses with optimum performance and linearity was observed (*R*^2^ > 0.998) between 5 and 50 μg mL^–1^ of digested protein. With regard to the LODs, all these peptides were detected until 2.5 μg mL^–1^ of digested protein, except for peptide [98-102] (5 μg mL^–1^).

**Table 1:** Peak Areas, Migration Times, and Percentage of Relative Standard Deviations (% RSD) of the Tryptic Peptides of α-syn Detected by (A) On-line Immobilized Enzyme Microreactor Capillary Electrophoresis-Mass Spectrometry (IMER-CE-MS) Analyzing a 10 μg mL^–1^ Recombinant α-syn Standard and On-line Aptamer Affinity Solid-Phase Extraction-IMER-CE-MS (AA-SPE-IMER-CE-MS) Analyzing (B) a 0.5 μg mL^–1^ Recombinant α-syn Standard, and (C) a Thermo-enriched Red Blood Cell (TE RBC) Lysate

		[M + *n*H]^*n*+^		(A) IMER-CE-MS				(B) AA-SPE-IMER-CE-MS				(C) AA-SPE-IMER-CE-MS TE RBC			
				10 μg mL^–1^ standard (*n* = 3)				0.5 μg mL^–1^ standard (*n* = 3)				lysate (*n* = 3)			
				peak area		migration time (min)		peak area		migration time (min)		peak area		migration time (min)	
peptide sequence^a^		*m*/*z*	*n*	mean (×10^7^)	% RSD	mean	% RSD	mean (×10^7^)	% RSD	mean	% RSD	mean (×10^7^)	% RSD	mean	% RSD
1	[1-6] MDVFMK^b^	385.6824, 770.3575	2,1	0.46	0.2	7.5	1.8	0.82	15.5	7.9	4.6	–	–	–	–
1′	[1-6]′ *N*-acetyl MDVFMK^c^	812.3682	1	–	–	–	–	–	–	–	–	1.10	1.5	10.5	0.5
2	[7-10] GLSK	404.2503	1	0.13	2.3	6.2	1.5	0.17	10.3	7.0	4.0	0.56	18.7	7.0	0.3
3	[11-12] AK	218.1499	1	0.07	5.6	5.3	1.8	0.02	8.2	6.4	3.5	0.12	6.6	6.6	0.3
4	[13-21] EGVVAAAEK	437.2374	2	0.31	5.3	8.0	1.9	0.34	5.5	8.3	5.2	0.47	11.5	8.0	0.8
5	[22-23] TK	248.1605	1	0.40	3.9	5.5	1.5	0.15	11.7	6.5	3.7	0.30	16.8	6.7	0.2
6	[24-32] QGVAEAAGK	415.7219	2	0.31	1.8	7.7	1.8	0.38	14.9	8.1	5.3	0.63	13.1	7.9	0.8
7	[35-43] EGVLYVGSK	476.2609	2	0.46	2.4	8.1	1.8	0.48	16.9	8.4	5.0	0.72	4.7	8.1	0.5
8	[46-58] EGVVHGVATVAEK	432.5700	3	0.44	3.8	7.0	1.3	0.47	15.3	7.5	4.2	0.58	5.4	7.4	0.4
9	[61-80] EQVTNVGGAVVTGVTAVAQK	643.3500	3	0.27	3.6	9.8	2.1	0.23	3.7	10.0	7.7	0.16	5.2	9.5	0.7
10	[81-96] TVEGAGSIAAATGFVK	739.8961	2	0.33	2.8	8.9	1.9	0.39	10.0	9.2	4.8	0.31	2.2	8.8	0.5
11	[98-102] DQLGK	560.3038	1	0.04	18.2	7.6	1.2	–	–	–	–	–	–	–	–
12	[103-140] NEEGAPQEGILEDMPVDPDNEAYEMPSEEGYQDYEPEA	1072.9489, 1430.2494	4, 3	–	–	–	–	–	–	–	–	–	–	–	–

a Single amino acids were not taken into account.

b Free α-syn was only detected in the recombinant protein.

c *N*-terminal acetylated α-syn was only detected in blood.

### Minimizing Nonspecific Retention in the Analysis of Blood α-syn by AA-SPE-CE-MS

Recently, we developed an AA-SPE-CE-MS methodology using AA-MBs blocked with ethanolamine for the analysis of intact α-syn in TE RBC lysates.^20^ As shown in Figure S-1A, *N*-acetylated α-syn, which is the main proteoform in blood,^20−22^ was detected, although nonspecific adsorption in the AA sorbent of ubiquitin and apolipoprotein A-I was also observed. Now, we have investigated new pretreatments both in the AA sorbent and in the TE RBC lysates to improve the detection of blood α-syn. Figure S-1B shows the analysis of a TE RBC lysate filtered through a 10 000 *M*_r_ cut-off (MWCO) centrifugal filter. In comparison to unfiltered samples (Figure S-1A), the intensity of the peak of *N*-acetylated α-syn increased due to the lower complexity of the sample matrix loaded. However, in both filtered and unfiltered samples, a similar amount of ubiquitin and apolipoprotein A-I was detected. To further decrease nonspecific protein adsorption in the AA sorbent, the use of bovine serum albumin (BSA) as blocking agent instead of ethanolamine was investigated. BSA is known for nonspecific binding reduction, and it is widely used for such a purpose in immunoaffinity assays.^32^Figure S-1C shows the analysis of a TE RBC lysate sample, filtered through a 10 000 MWCO filter, using AA-MBs blocked with BSA in AA-SPE-CE-MS. Compared to AA-MBs blocked with ethanolamine (Figure S-1B), the peak area of ubiquitin and apolipoprotein A-I decreased 59% and 34%, respectively. Furthermore, the peak area of *N*-acetylated α-syn increased 45%, due to the minimized nonspecific retention of the interfering proteins. This explanation is also supported by the fact that for the analysis of recombinant α-syn standard by AA-SPE-CE-MS no differences were found using AA-MBs blocked with ethanolamine or BSA and, in both cases, the LOD was 0.2 μg mL^–1^ as in our previous study,^20^ a value 100 times lower compared to CE-MS (20 μg mL^–1^). In view of the better performance of the AA-MBs blocked with BSA for the analysis of TE RBC lysates, this improved AA sorbent was used in the subsequent experiments.

### Optimization of the Bottom-Up Analysis of α-syn by AA-SPE-IMER-CE-MS

In order to further decrease the LOD of α-syn, detecting peptide biomarkers instead of the intact protein, and better characterization of endogenous α-syn, including localization of PTMs, we investigated AA-SPE-IMER-CE-MS.

The starting point for the optimization of the AA-SPE-IMER-CE-MS methodology was the optimized conditions for AA-SPE-CE-MS and IMER-CE-MS. Preliminary experiments demonstrated that coupling of 0.7 cm microcartridges and microreactors excessively increased backpressure. Therefore, to avoid current instability and breakdowns during electrophoretic separations, the length of both microdevices was reduced to 0.5 cm.

**Figure 3:**
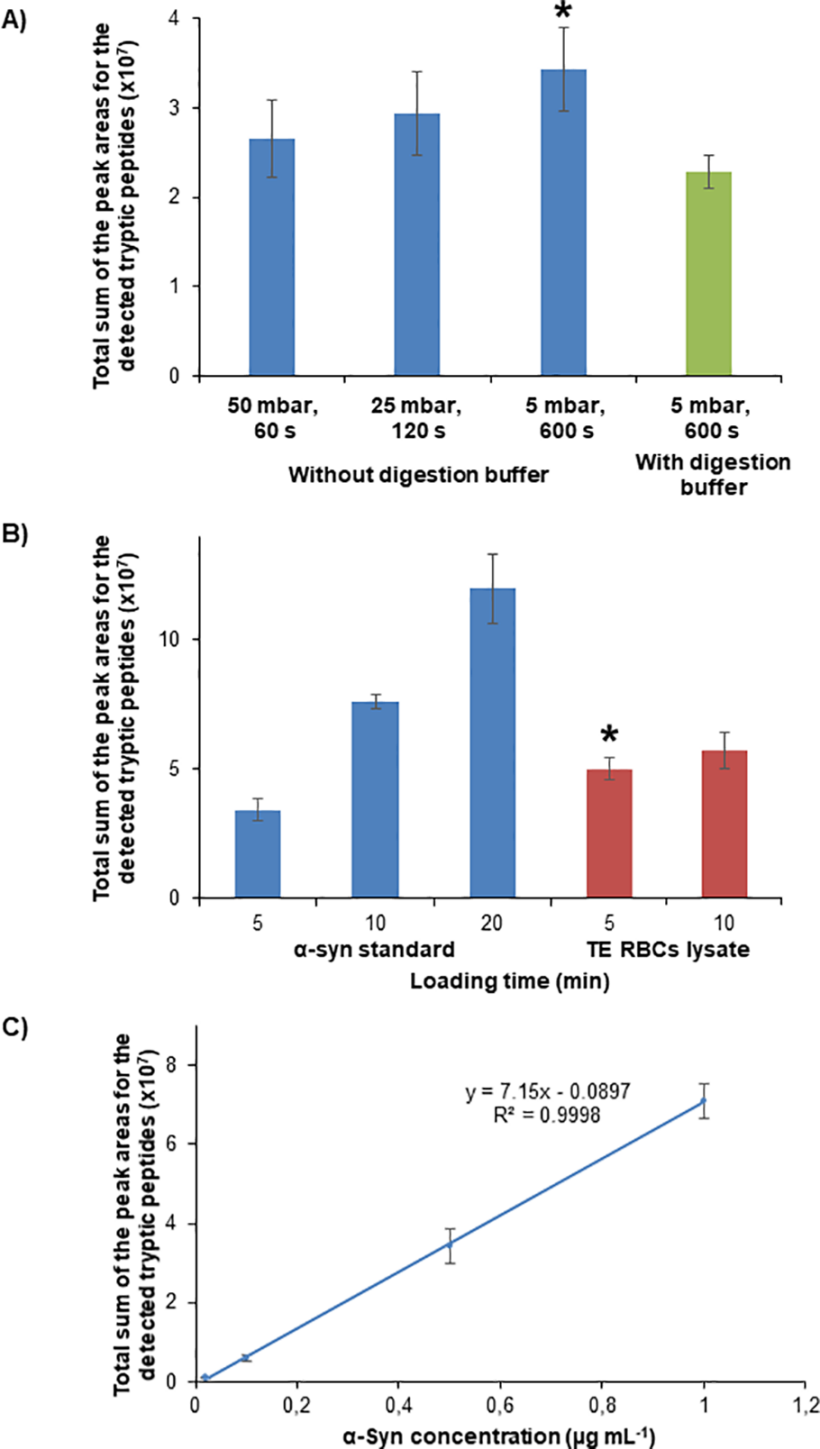
Plot of the total sum of peak areas for the detected tryptic peptides vs (A) mobilization/digestion velocity by pushing the eluent with BGE without digestion buffer (10 mM NH_4_HCO_3_, pH 7.9) (blue) or between two plugs of digestion buffer (green) (loading, 0.5 μg mL^–1^ recombinant α-syn standard in water at 930 mbar for 5 min); (B) loading time for a 0.5 μg mL^–1^ recombinant α-syn standard in water (blue) and a thermo-enriched red blood cell (TE RBC) lysate sample filtered through a 10 000 MWCO filter (red) (mobilization/digestion velocity, 5 mbar for 600 s by pushing the eluent with BGE); and (C) concentration of the loaded recombinant α-syn standard (loading, 930 mbar for 5 min; mobilization/digestion velocity, 5 mbar for 600 s by pushing the eluent with BGE). Optimized conditions are indicated with an asterisk. Other conditions: microcartridge (0.5 cm *L*_T_ × 250 μm i.d.), microreactor (0.5 cm *L*_T_ × 250 μm i.d.), separation capillary (72 cm *L*_T_ × 75 μm i.d.) and BGE (50 mM HAc and 50 mM HFor, pH = 2.3). Sample dissolved in water. Eluent (100 mM NH_4_OH (pH 11.2)) injected at 50 mbar for 40 s. Preconcentration-digestion-separation temperature 37 °C and separation voltage +25 kV. Data extraction considering the *m*/*z* of the most abundant molecular ions (Table 1) and a window of ±20 ppm. All measurements were performed in triplicate (standard deviation is given as error bars).

In immunoaffinity or in aptamer affinity SPE-CE-MS, the elution of the target protein is typically fast and takes place after applying the voltage for the electrophoretic separation or, in some cases to improve peak shape and repeatability, after pushing the eluent with BGE at a small pressure (e.g., 50 mbar) before the separation.^17,20,27,28^ In IMER-CE-MS, the small volume of protein to be digested is also mobilized pushing at a very small pressure (e.g., 5 mbar) to maximize the contact time with the immobilized enzyme before applying the voltage for the separation.^29^ The elution-mobilization-digestion step was investigated in AA-SPE-IMER-CE-MS loading a 0.5 μg mL^–1^ recombinant α-syn standard for 5 min, injecting the eluent of 100 mM NH_4_OH (pH 11.2) at 50 mbar for 40 s (∼220 nL^31^, i.e., ∼5 cm) (eluent was optimized in our previous study by AA-SPE-CE-MS^20^) and mobilizing this eluent by pushing with BGE at different velocities for digestion (50 mbar, 60 s; 25 mbar, 120 s and 5 mbar, 600 s; in all cases, ∼325 nL,^31^ i.e., ∼7 cm). As can be seen in Figure 3A, the highest repeatability and peak area for the peptides was obtained at the smallest velocity (5 mbar, 600 s), similarly to the IMER-CE-MS results of our previous study.^29^

It is important to note that probably a pH gradient was generated during eluent mobilization due to the eluent-BGE contact, and these conditions allowed an appropriate enzyme activity and efficient digestion. Therefore, it was not necessary to perform protein digestion as in IMER-CE-MS, sandwiching the eluent between two plugs of digestion buffer (10 mM NH_4_HCO_3_, pH 7.9). Under these “sandwich” conditions in AA-SPE-IMER-CE-MS, peak area for the peptides decreased (Figure 3A) and separations deteriorated, probably due to decreased elution in the AA sorbent and anti-stacking effects in the boundary between the digestion buffer plug and the eluent.

Once the mobilization and digestion conditions were optimized, the sample loading time was investigated introducing a 0.5 μg mL^–1^ α-syn standard at 930 mbar from 5 to 20 min. As can be seen in Figure 3B, the maximum value for the total sum of peak areas for the detected tryptic peptides was obtained loading the α-syn standard for 20 min (blue bars). However, as will be discussed in the following section, the best results for blood α-syn were achieved loading the TE RBC lysate for 5 min (red bars). Therefore, to be consistent with the conditions applied for the analysis of blood samples, the method quality parameters with standards were investigated for a sample loading time of 5 min.

**Figure 4:**
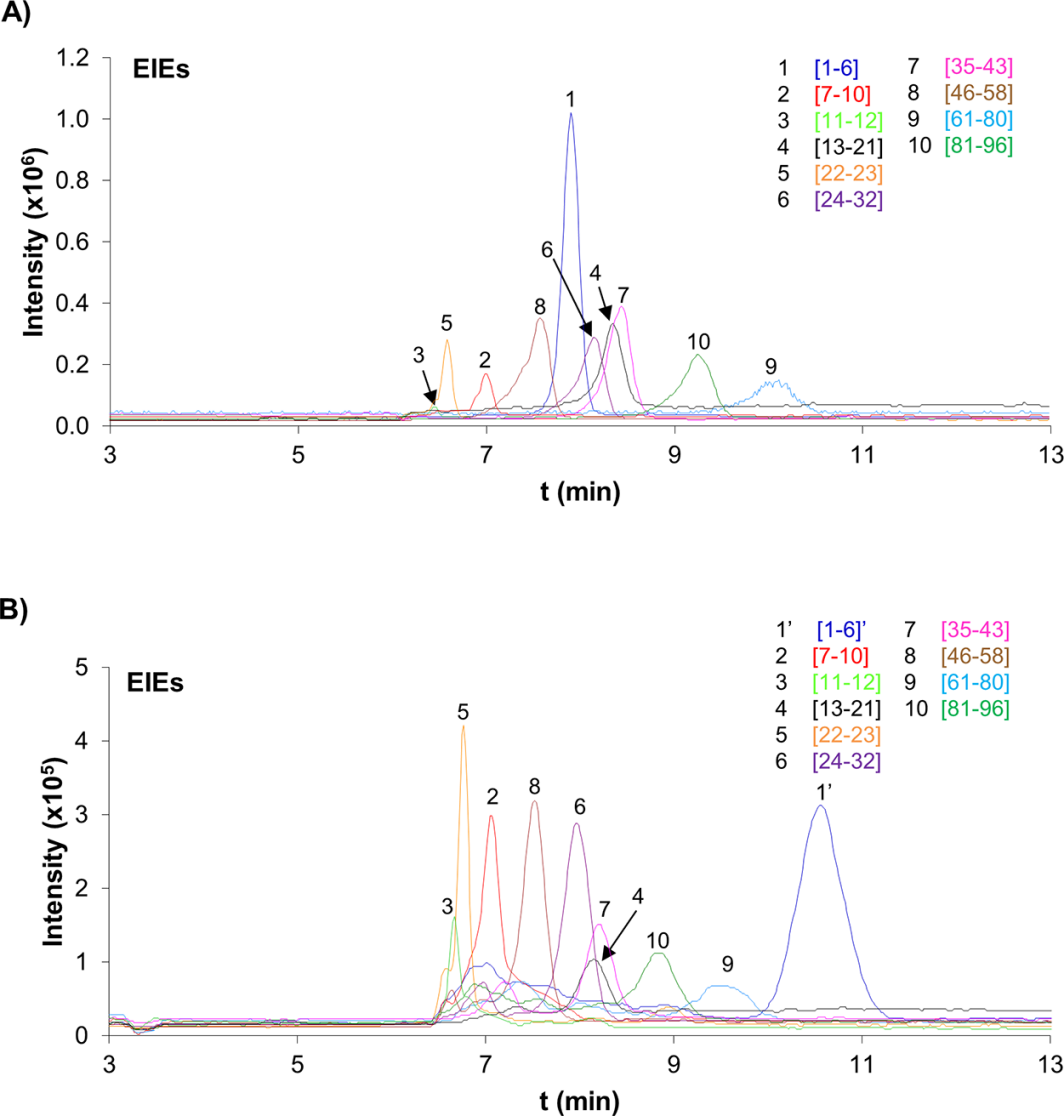
Analysis of α-syn by AA-SPE-IMER-CE-MS. EIEs for (A) a 0.5 μg mL^–1^ recombinant α-syn standard and (B) a thermo-enriched red blood cell (TE RBC) lysate sample filtered through a 10 000 MWCO filter. Conditions: microcartridge (0.5 cm *L*_T_ × 250 μm i.d.), microreactor (0.5 cm *L*_T_ × 250 μm i.d.), separation capillary (72 cm *L*_T_ × 75 μm i.d.), and BGE (50 mM HAc and 50 mM HFor, pH = 2.3). Sample dissolved in water and loaded at 930 mbar for 5 min. Eluent (100 mM NH_4_OH (pH 11.2)) injected at 50 mbar for 40 s and pushed with BGE at 5 mbar for 600 s. Preconcentration-digestion-separation temperature 37 °C and separation voltage +25 kV. Data extraction considering the *m*/*z* of the most abundant molecular ions (Table 1) and a window of ±20 ppm.

Figure 4A shows the EIEs and Table 1B the peak area and migration time values as well as the percentages of relative standard deviation (% RSD, *n* = 3), for a 0.5 μg mL^–1^ α-syn standard analyzed by AA-SPE-IMER-CE-MS in the selected conditions. At this protein concentration, 10 out of 12 expected peptides were detected (83.3% of coverage), and repeatability using a single modified capillary was adequate (% RSD (*n* = 3) ranged from 3.5 to 7.7% for peak areas and 3.7 to 16.9% for migration times (Table 1B), that is to say, 4.8% and 12.7% for migration times and total sum of peptide peak areas). Capillary-to-capillary repeatability was evaluated using three different modified capillaries (*n* = 9/3 capillaries). Repeatability on migration time and total sum of peptide peak areas increased only slightly compared to the values obtained for a single capillary (% RSD = 5.2% and 17.4%, respectively), as expected because modified capillaries were homemade. The modified capillaries could be reused up to 10 times with optimum performance, similarly to IMER-CE-MS.

As can be seen in Figure 3C, the method was satisfactorily linear (*R*^2^ > 0.999) between 0.02 and 1 μg mL^–1^. At 0.02 μg mL^–1^, 8 out of 12 expected peptides were detected (66.7% of coverage), while no peptides were detected when decreasing the concentration of the α-syn standard to 0.01 μg mL^–1^. Therefore, the LOD was improved around 125 and 10 times compared to IMER-CE-MS (2.5 μg mL^–1^) and AA-SPE-CE-MS (0.2 μg mL^–1^), respectively.

### Analysis of α-syn in Blood Samples

The AA-SPE-IMER-CE-MS method was applied to the analysis of TE RBC lysate samples. The sample loading time was investigated introducing at 930 mbar a TE RBC lysate sample filtered through a 10 000 MWCO filter. As can be seen in Figure 3B, when loading the TE RBC lysate for more than 5 min, the sorbent was saturated and there was no expected increase of peak areas for the peptides of blood α-syn (red bars). A *t* test (with confidence of 95%) was performed to compare the mean of the total sum of the peak areas for the tryptic peptides detected when loading for 5 and 10 min, and no significant difference was found. The rapid saturation of the sorbent compared to the standards is probably due to the higher sample matrix complexity. To minimize sample consumption and reduce the total analysis time, a sample loading time of 5 min was selected.

Figure 4B shows the EIEs and Table 1C the peak area and the migration time values as well as the percentages of relative standard deviation (% RSD, *n* = 3), for the analysis of a TE RBC lysate sample by AA-SPE-IMER-CE-MS. A total of 10 out of 12 expected peptides were detected (83.3% of coverage), repeatability was adequate with a % RSD (*n* = 3) ranging from 1.5 to 18.7% for peak areas and 0.2 to 0.8% for migration times, and the modified capillary could be reused up to 10 analyses as with the standards.

As shown in Table 1B,C, the migration times of most of the peptides were similar in standards and biological samples. A *t* test (with confidence of 95%) was performed to compare the migration times for all the peptides, except for peptide [1-6], in standards and TE RBC lysate samples and no significant differences were found. This confirmed that loading a complex matrix sample did not modify the inner wall of the separation capillary. However, the peptide [1-6] migrated last in the TE RBC lysate electropherogram (Figure 4B) due to the acetylation of the *N*-terminal amino group in the main proteoform of blood α-syn, an uncharged PTM that decreases the charge and increases the *M*_r_ of the peptide (Table 1). The detection of most of the expected peptides proves that the developed AA-SPE-IMER-CE-MS method allows the detailed characterization of blood α-syn, including its main PTM.^22^ In the future, the developed method could be applied to the analysis of other endogenous α-syn proteoforms,^21,23^ such as those found in cerebrospinal fluid or in the Lewy bodies isolated from the brain of patients with different synucleinopathies to screen for other characteristic PTMs that could be targeted as disease biomarkers.

## Concluding Remarks

We have developed a fully integrated valve-free on-line AA-SPE-IMER-CE-MS method for purification, preconcentration, tryptic digestion, separation, and characterization of blood α-syn in less than 35 min. Under the optimized conditions with recombinant α-syn standards, the repeatability was adequate (at 0.5 μg mL^–1^,% RSD ranged from 3.7 to 16.9% for peak areas and 3.5 to 7.7% for migration times) and the modified capillary could be reused up to 10 analyses with optimum performance. The LOD was 0.02 μg mL^–1^ of digested protein, with a good protein coverage (66.7%), hence 125 and 10 times lower than by IMER-CE-MS (2.5 μg mL^–1^) and AA-SPE-CE-MS (0.2 μg mL^–1^), respectively. Regarding the analysis of TE RBC lysate samples, an 83.3% protein coverage of blood α-syn was achieved, enabling the detailed characterization of the protein and localization of the most abundant PTM (i. e., *N*-terminal acetylation). The presented method could be easily adapted to analyze other protein biomarkers or biopharmaceuticals using other specific aptamers and trypsin as well as other specific proteolytic enzymes to achieve complementary sequence and PTMs coverage. In addition, sensitivity could be further enhanced through targeted MS-MS measurements with state-of-the-art mass spectrometers.

## Supporting Information Available

Additional experimental details: optimized MS parameters and procedures to measure the quality parameters and additional extracted ion electropherograms (EIEs) for TE RBC lysates using AA-SPE-CE-MS (Figure S-1) (PDF)
