# Supplementary material for: On-line Coupling of Aptamer Affinity Solid-Phase Extraction and Immobilized Enzyme Microreactor Capillary Electrophoresis-Mass Spectrometry for the Sensitive Targeted Bottom-Up Analysis of Protein Biomarkers
Source: Anal Chem. 2022 May 2;94(19):6948–56. doi: 10.1021/acs.analchem.1c03800 (PMC9118193; doi:10.1021/acs.analchem.1c03800)
Supplement: Supplementary file 1 — ac1c03800_si_001.pdf [file ac1c03800_si_001.pdf]

Supporting Information

On-line coupling of aptamer affinity solid-phase extraction and immobilized enzyme microreactor capillary electrophoresis-mass spectrometry for the sensitive targeted bottom-up analysis of protein biomarkers

Hiba Salim[#], Roger Pero-Gascon[#], Estela Giménez and Fernando Benavente[*]

Department of Chemical Engineering and Analytical Chemistry, Institute for Research on Nutrition and Food Safety (INSA·UB), University of Barcelona, Barcelona 08028, Spain

[*] Corresponding author: fbenavente@ub.edu (F. Benavente, PhD)

Tel: (+34) 934035423, Fax: (+34) 934021233

[#] H.S. and R.P. contributed equally to this paper.

Table of contents

MS parameters S-2

Quality parameters S-2

Figure S-1. AA-SPE-CE-MS of TE RBC lysates S-3

MS parameters

The TOF mass spectrometer was operated under optimized conditions in positive mode using the following parameters: capillary voltage 4000 V, drying temperature 200 ºC, drying gas flow rate 4 L min^{-1}, nebulizer gas 7 psig, fragmentor voltage 190 V, skimmer voltage 60 V, OCT 1 RF Vpp voltage 300 V. Data were collected in profile at 1 spectrum/s between 100 and 3200 m/z, with the mass range set to high resolution mode (4 GHz).

Quality parameters

All quality parameters were calculated from data obtained by measuring peak area and migration time from the extracted ion electropherogram (EIE) of α-syn tryptic peptides considering the *m/z* of the most abundant molecular ions (Table 1) and a window of ±20 ppm. Repeatability was evaluated as the percent relative standard deviation (%RSD) of peak area and migration time. The LOD was obtained by analyzing low-concentration standard solutions of recombinant α-syn (close to the LOD level, as determined from a S/N=3). Linearity range was established by analyzing recombinant α-syn standards at concentrations from 2.5 to 50 $\mu g \cdot mL^{-1}$ for IMER-CE-MS and 0.02 to 1 $\mu g \cdot mL^{-1}$ for AA-SPE-IMER-CE-MS. The lifetime of IMER-CE-MS capillaries was investigated by repeatedly analyzing a 10 $\mu g\ mL^{-1}$ recombinant α-syn standard. The lifetime of AA-SPE-IMER-CE-MS capillaries was investigated by repeatedly analyzing a 0.5 $\mu g \cdot mL^{-1}$ recombinant α-syn standard and a TE RBC lysate sample. In both cases, the modified capillaries were discarded when the total sum of peak areas of α-syn peptides in the EIEs decreased more than 25% compared to the mean value of the first three analyses with the capillary under consideration.

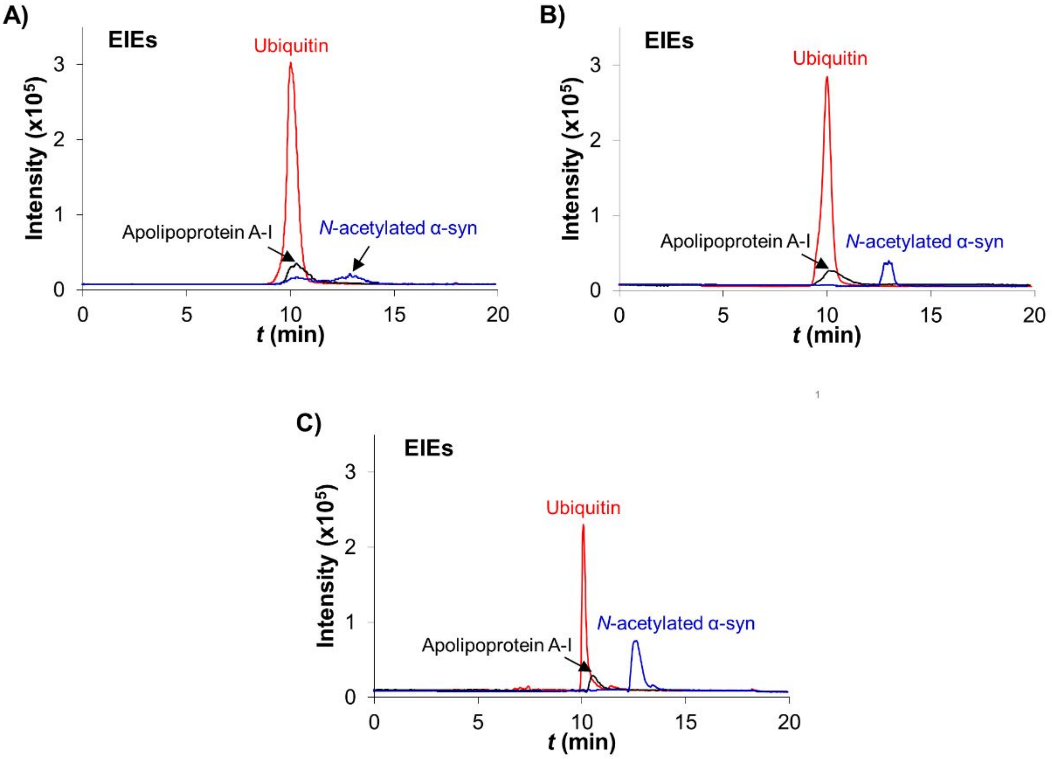

Figure S-1. Extracted ion electropherograms (EIEs) for ubiquitin (M_r = 8,565; *m/z* 1071.6019, 1224.5440, 1428.4668, i.e. z = +8, +7, +6), apolipoprotein A-I (M_r = 28,078; *m/z* 851.8658, 878.4552, 906.7599, i.e. z = +33, +32, +31) and N-acetylated α-syn (M_r = 14,502; *m/z* 907.3860, 967.8113, 1036.8687, i.e. z = +16, +15, +14) in thermo-enriched red blood cell (TE RBC) lysates. AA-SPE-CE-MS using AA-MBs blocked with ethanolamine: **(A)** unfiltered sample and **(B)** a sample filtered through a 10,000 M_r cut-off (MWCO) cellulose acetate centrifugal filter. **(C)** AA-SPE-CE-MS using AA-MBs blocked with bovine serum albumin (filtered sample).
